# Echocardiographic changes following dual-chamber pacemaker implantation at a Malaysian tertiary heart center

**DOI:** 10.1186/s44348-026-00077-6

**Published:** 2026-07-06

**Authors:** Si Ling Soh, Ian J. Y. Wee, Rowina Lynne Murray binti Jeffery Murray, Suraya Hani Kamsani, Aslannif Roslan, Azlan Hussin

**Affiliations:** 1 National Heart Institute, Kuala Lumpur, Malaysia; 2 Health Services Research Unit, Singapore General Hospital, Singapore, Singapore; 3 Duke-NUS Medical School, Singapore, Singapore

**Keywords:** Dual chamber pacemaker, Echocardiography, Strain imaging

## Abstract

**Background:**

Dual-chamber pacemaker implantation restores atrioventricular synchrony, but it might be associated with alterations in ventricular mechanics and valvular function. The echocardiographic effects of dual-chamber pacing in contemporary practice remain incompletely characterized.

**Methods:**

We conducted a retrospective single-center study of patients who underwent dual-chamber pacemaker implantation and had transthoracic echocardiography performed before and after implantation in 2022. Changes in left ventricular ejection fraction (LVEF), ventricular and atrial functional parameters, strain-derived measures, and tricuspid regurgitation (TR) severity were assessed using paired statistical analyses.

**Results:**

In total, 57 patients were included. The mean LVEF declined from 59.6% before implantation to 55.8% after implantation (mean change, − 3.76%; 95% confidence interval [CI], − 7.45 to 0.08; *P* = 0.051). Among the strain-based measures, right ventricular global longitudinal strain (RVGLS) worsened by a mean of 3.10% (95% CI, 0.73 to 5.48; *P* = 0.012), and left atrial conduit strain (LAScd) declined by 3.74% (95% CI, 3.11 to 4.37; *P* = 0.032) following pacemaker implantation. Stuart-Maxwell testing showed no significant overall shift in TR severity following pacemaker implantation (χ^2^ = 24.94, *P* = 0.678). The left atrial volume index decreased significantly (38.98 ± 17.16 mL/m^2^ vs. 34.38 ± 12.65 mL/m^2^, *P* = 0.023), but it showed no correlation with LAScd at baseline (*ρ* =  − 0.183, P = 0.265) or with changes in LAScd over time (*ρ* = 0.160, *P* = 0.350). In regression analyses, increasing age was independently associated with greater decline in LAScd (β =  − 0.323 per year; 95% CI, − 0.494 to − 0.152; *P* < 0.001). A high pacing burden (> 20%) was not associated with changes in LVEF, LAScd, or RVGLS.

**Conclusions:**

Dual-chamber pacemaker implantation was associated with early deterioration in myocardial deformation, particularly RVGLS and LAScd, despite preserved conventional systolic parameters. These functional changes were not accompanied by structural remodeling and were independent of TR progression, supporting a mechanism related to pacing-induced dyssynchrony and altered ventricular-atrial coupling. Strain imaging could offer incremental value in detecting early subclinical dysfunction in this population.

**Supplementary Information:**

The online version contains supplementary material available at 10.1186/s44348-026-00077-6.

## Introduction

Permanent pacemaker implantation remains a cornerstone therapy for bradyarrhythmias and atrioventricular conduction disease, with dual-chamber pacing widely adopted to preserve atrioventricular synchrony and optimize hemodynamics [1]. Despite its physiological advantages over single-chamber ventricular pacing, chronic right ventricular (RV) pacing has been associated with abnormal ventricular activation patterns that might adversely affect cardiac structure and function over time [2–4].

RV apical pacing produces an electrical activation sequence similar to left bundle branch block, resulting in electrical and mechanical dyssynchrony [3]. Evidence demonstrates that such dyssynchrony can lead to adverse left ventricular (LV) remodeling, reductions in LV ejection fraction (LVEF), and the development of pacing-induced cardiomyopathy in susceptible patients [3, 4]. Deterioration in LV systolic function has been observed with higher cumulative RV pacing burdens, even among individuals with preserved baseline LVEF [3–5]. In addition to LV systolic function, pacemaker implantation might also affect right-sided cardiac structures and valvular competence. Transvenous pacemaker and defibrillator leads can cause severe, symptomatic tricuspid regurgitation (TR) through mechanical interference with the tricuspid valve apparatus [6], resulting in right heart failure, atrial arrhythmias, and increased cardiovascular-associated mortality [6, 7].

Advances in echocardiography, particularly deformation imaging using strain analyses, have improved the detection of subclinical myocardial dysfunction. LV and RV global longitudinal strain (GLS), as well as phasic left atrial (LA) strain, provide incremental information beyond conventional parameters such as LVEF and tricuspid annular plane systolic excursion (TAPSE) [8, 9]. Strain-derived measures can detect early pacing-related myocardial dysfunction before overt declines in LVEF become apparent.

In Malaysia, dual-chamber pacemakers continue to be implanted frequently, but their short-term echocardiographic outcomes have not been comprehensively evaluated. Therefore, in this study, we evaluate paired echocardiographic changes following dual-chamber pacemaker implantation at a Malaysian tertiary heart center. By assessing ventricular and atrial function, strain-derived parameters, and TR severity before and after implantation, we sought to characterize the early echocardiographic effects of dual-chamber pacing in our population.

## Methods

### Ethics statement

This study was approved by the Institut Jantung Negara Research Ethics Committee (No. IJNREC/818/2025). Informed consent was waived due to the use of deidentified data and the retrospective nature of the study. The study was performed in accordance with the Declaration of Helsinki.

### Study design and population

This retrospective observational study was conducted at a single tertiary heart center in Malaysia. Consecutive adult patients who underwent dual-chamber pacemaker implantation in 2022 were identified. Patients were included if they had paired 2D transthoracic echocardiographic examinations performed before and after implantation (within 6 months of the procedure). Patients without complete paired echocardiographic data were excluded. In this cohort, follow-up studies were conducted within a relatively narrow time window, predominantly between 5 and 6 months. Given that limited variability, sensitivity analyses with different follow-up intervals were not performed because they would not meaningfully alter the results.

### Echocardiographic assessment

Two-dimensional speckle-tracking strain analyses were performed offline using vendor-independent software (2D Cardiac Performance Analysis; TomTec Imaging Systems, Phillips) according to institutional protocols. All echocardiographic acquisitions and analyses were performed by a single experienced operator using a standardized imaging protocol. LV systolic function was assessed using the LVEF, calculated using the biplane method of disk summation (modified Simpson rule) [10]. TAPSE and RV systolic velocity (RV S′) were measured from the RV-focused apical four-chamber view. TAPSE was obtained using the M-mode with the cursor aligned through the lateral tricuspid annulus, and RV S′ was measured using pulsed-wave tissue Doppler imaging at the lateral tricuspid annulus. Myocardial strain was assessed using GLS where available: RVGLS, RV free wall strain (RVFWS), and LVGLS. LA function was assessed using the LA reservoir strain (LASr), LA conduit strain (LAScd), and LA contractile strain (LASct). Specifically, LA strain was assessed using 2D speckle-tracking analyses from both the apical four-chamber and apical two-chamber views, with biplane averaging of all segments. A standard 12-segment model was used for those analyses. The strain measurements were averaged over three cardiac cycles, with the onset of the P-wave used as the zero-reference point. The LA volume index (LAVI) was assessed as a measure of LA structural remodeling. LA volumes were obtained using the biplane area-length method from apical four- and two-chamber views and indexed to body surface area in accordance with current echocardiographic guidelines [11]. At our center, TR severity is assessed qualitatively based on standard clinical echocardiographic interpretation. For the purposes of our analyses in this study, TR severity was categorized into four grades (trivial/trace, mild, moderate, and severe) in accordance with the current American Society of Echocardiography (ASE) guidelines [11]. Patients with no TR were graded in a fifth category as “none.” In some patients, TR severity could not be reliably assessed due to suboptimal echocardiographic image quality, particularly inadequate visualization of the tricuspid valve and regurgitant jet. Those patients were excluded from the paired TR transition analyses.

### Outcomes

The primary outcome was the change in LVEF in echocardiography taken before and after implantation. The secondary outcomes were changes in other echocardiographic measures of RV and atrial function and changes in the distribution of TR severity after pacemaker implantation. For continuous echocardiographic outcomes, paired differences were calculated as post-implant value minus pre-implant value using the original signed measurements. For strain parameters reported as negative values (e.g., LVGLS, RVGLS, and RVFWS), a positive change reflects a shift toward less negative strain values and was thus interpreted as worsening myocardial deformation.

### Statistical analysis

Continuous variables are presented as the mean ± standard deviation, unless otherwise stated. Paired echocardiographic measurements obtained before and after pacemaker implantation were compared within individuals.

For continuous echocardiographic outcomes, paired differences were calculated as post-implant value minus pre-implant value. The normality of paired differences for the primary outcome was assessed using a visual inspection of histograms and the Shapiro–Wilk test. Given the sample size and approximate normal symmetry of distributions, paired t-tests were used to compare the pre- and post-implant values of the echocardiographic measures, and results are reported as the mean change with corresponding 95% confidence intervals (CIs).

Device interrogation data were reviewed to obtain the pacing mode and cumulative RV pacing burden. The RV pacing burden was dichotomized into low (≤ 20%) and high (> 20%) based on clinically relevant thresholds [4, 12]. To evaluate the potential influence of pacing on echocardiographic parameters, the RV pacing burden was included as a covariate in the regression analyses.

To identify factors associated with changes in the echocardiographic parameters, linear regression analyses were performed, with the change in LVEF and strain parameters as the dependent variables. The covariates were baseline demographic and clinical characteristics (age, sex, and the comorbidities of diabetes mellitus, hypertension, dyslipidemia, and chronic kidney disease) and the RV pacing burden described above. Regression coefficients (β) with 95% CIs are reported.

Changes in TR severity were evaluated as a paired ordinal variable using the Stuart-Maxwell test of marginal homogeneity. In addition, transitions between severity categories are summarized using a transition matrix to describe the direction and magnitude of change across the full spectrum of TR severity.

To evaluate the relationship between structural and functional atrial parameters, correlations between the baseline LAVI and LAScd were assessed using Spearman rank correlation coefficients. In addition, exploratory analyses were performed to assess correlations between changes in LAVI and changes in LAScd over time.

Analyses were conducted using available paired data for each outcome, and denominators are reported accordingly. Missing echocardiographic data, particularly for strain parameters, were primarily due to suboptimal image quality that precluded a reliable speckle-tracking analysis. No imputation was performed for missing data. However, to assess potential selection bias, baseline characteristics were compared between patients with and without available strain data.

All statistical tests were two-sided, and a *P*-value of < 0.05 was considered statistically significant. Statistical analyses were performed using Stata ver. 18.5 (StataCorp).

## Results

### Study population

In total, 57 patients were included in the analysis. They had a mean age 58.5 ± 22.2 years, underwent dual-chamber pacemaker implantation, and had paired pre- and post-implantation echocardiographic assessments. Most of the patients were male (*n* = 32, 56.1%) and predominantly Malays (*n* = 34, 59.6%). Dual-chamber pacemaker implantation was done for various indications, most commonly for sick sinus syndrome (*n* = 40, 70.2%), and 24 (42.1%) had a pacing burden > 20%. The most common pacing mode was the DDD mode (dual-chamber pacing and sensing with dual response; *n* = 21, 36.8%), followed by the MVPR mode (managed ventricular pacing mode; *n* = 18, 31.6%) and the DDDR mode (rate-responsive DDD; *n* = 15, 26.3%). The VVIR mode (ventricular pacing and sensing with rate responsiveness mode) was used in a small minority of patients (*n* = 3, 5.3%). The mean interval between pacemaker implantation and post-implantation echocardiography was 5.2 ± 0.3 months. Baseline characteristics were comparable between patients with and without available strain data (Table S1). A summary of the baseline characteristics can be found in Table 1.

**Table 1:** Baseline characteristics (*n* = 57)

Characteristic	Value
Age (yr)	58.5 ± 22.2
Male sex	32 (56.1)
Race	
Chinese	16 (28.1)
Indian	7 (12.3)
Malay	34 (59.6)
Diabetes mellitus	15 (26.3)
Hypertension	30 (52.6)
Hypercholesterolemia	14 (24.6)
Chronic kidney disease	5 (8.8)
Indication	
Sick sinus syndrome	40 (70.2)
Atrioventricular block	17 (29.8)
Pacing burden > 20%	24 (42.1)
Pacing mode	
DDD	21 (36.8)
DDDR	15 (26.3)
MVPR	18 (31.6)
VVIR	3 (5.3)
Alive at discharge	57 (100)
Alive at 1-yr follow-up	55 (96.5)

Values are presented as mean ± standard deviation or number (%)

### Changes in LV systolic function

**Fig. 1:**
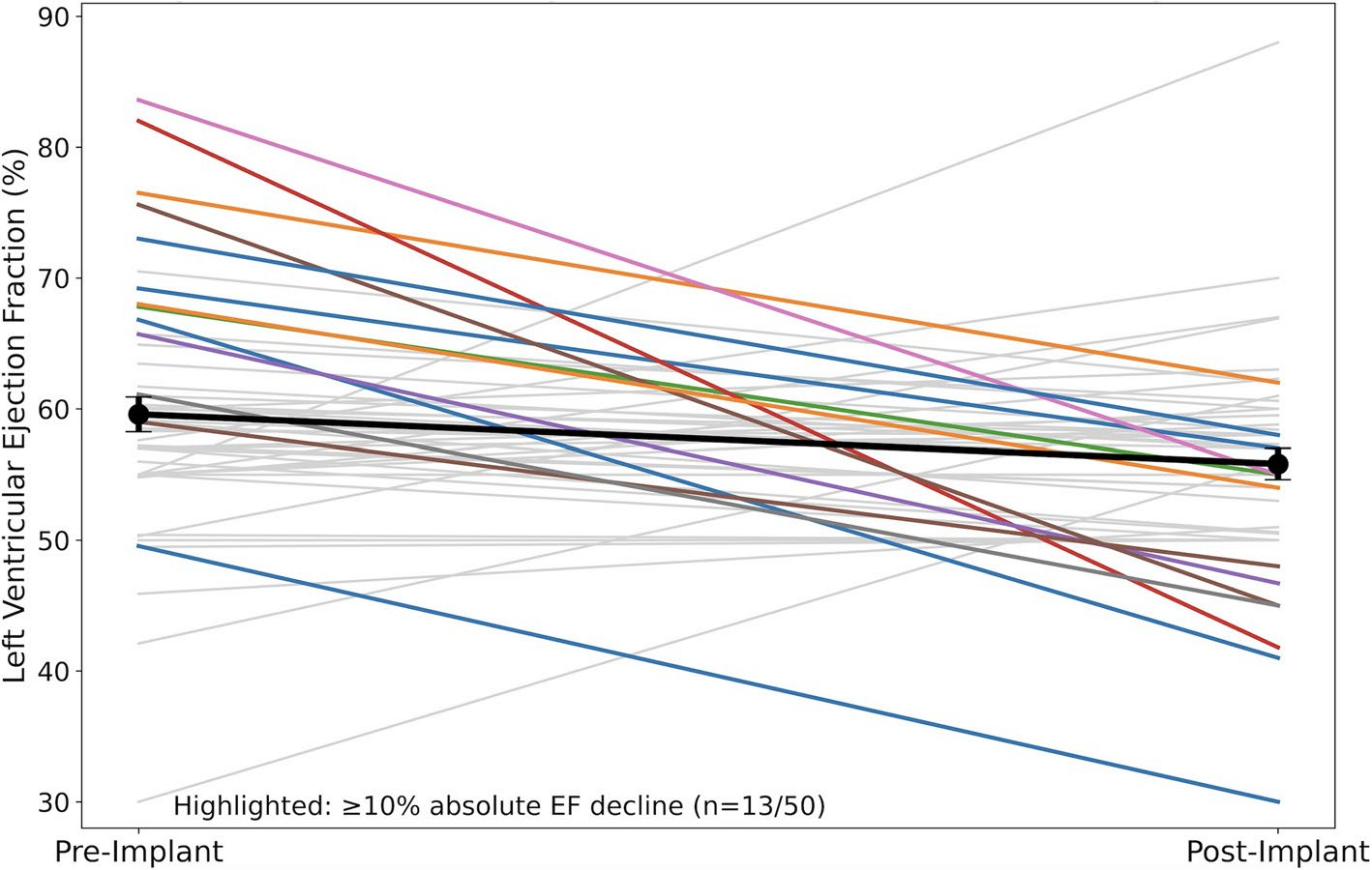
Paired changes in left ventricular ejection fraction (LVEF) before and after dual-chamber pacemaker implantation. The gray lines denote individual trajectories; colored lines indicate patients with ≥ 10% absolute decline in LVEF. The black line shows the mean change with 95% confidence intervals

The paired echocardiographic assessments demonstrated a nonsignificant reduction in LVEF after pacemaker implantation (Fig. 1). Mean LVEF decreased from 59.6% before implantation to 55.8% after implantation, corresponding to a mean change of − 3.76% (95% CI, − 7.45 to 0.08; *P* = 0.051) (Table 2). Individual patient trajectories demonstrated heterogeneity in the LVEF response, with most patients exhibiting modest changes and a small subset showing more pronounced declines (Fig. 1). In baseline-adjusted univariate linear regression analyses, no baseline clinical variables were significantly associated with the change in LVEF following pacemaker implantation (Table 3).

**Table 2:** Summary of outcomes before and after dual-chamber pacemaker implantation

Variable	No. of patients	Before implantation	After implantation	Change (95% CI)	*P*-value
Ejection fraction (%)	50	59.6 ± 1.3	55.8 ± 1.2	–3.76 ± 1.91 (–7.45 to 0.08)	0.051
TAPSE (cm)	48	1.98 ± 0.07	1.90 ± 0.06	–0.08 ± 0.07 (–0.22 to 0.06)	0.297
RV S’ (cm/sec)	47	11.39 ± 0.40	10.64 ± 0.44	–0.75 ± 0.39 (–1.51 to 0.01)	0.059
RVGLS (%)	36	–16.95 ± 0.94	–13.85 ± 1.12	3.10 ± 1.17 (0.73 to 5.48)	0.012
RVFWS (%)	36	–19.81 ± 1.31	–16.76 ± 1.46	3.05 ± 9.55 (–0.17 to 6.27)	0.062
RV basal dimension (cm)	44	3.44 ± 0.58	3.60 ± 0.66	0.16 ± 0.70 (–1.21 to 1.53)	0.135
RV mid dimension (cm)	44	2.91 ± 0.59	2.87 ± 0.72	–0.03 ± 0.75 (–1.50 to 1.44)	0.780
LVGLS (%)	42	–15.06 ± 5.01	–13.54 ± 4.01	1.52 ± 4.60 (–0.13 to 2.91)	0.118
LASr (%)	37	22.44 ± 11.64	19.18 ± 10.58	− 3.26 ± 15.09 (–8.29 to 1.77)	0.197
LAScd (%)	36	–16.43 ± 1.56	–12.69 ± 1.16	3.74 ± 1.68 (3.11 to 4.37)	0.032
LASct (%)	36	–7.48 ± 1.21	–8.71 ± 1.01	–1.23 ± 4.26 (− 3.48 to 1.02)	0.282
LAVI (mL/m^2^)	48	38.98 ± 17.16	34.38 ± 12.65	–4.60 ± 15.23 (–7.30 to –1.90)	0.023
TR severity (No. of patients)	50	50	50		0.678
None		14	0	–14	
Trivial		23	17	–6	
Mild		4	13	9	
Moderate		6	10	4	
Severe		3	10	7	

Values are presented as mean ± standard deviation, unless otherwise indicated. Shapiro–Wilk test for normal data: *P* = 0.08476. Histogram: normal distribution with no significant degree of skewness

*CI* confidence interval, *LAScd* left atrial conduit strain, *LASct* left atrial contractile strain, *LASr* left atrial reservoir strain, *LAVI* left atrial volume index, *LVGLS* left ventricular global longitudinal strain, *RV* right ventricular, *RVFWS* right ventricular free wall strain, *RVGLS* right ventricular global longitudinal strain, *TAPSE* tricuspid annular plane systolic excursion, *TR* tricuspid regurgitation

**Table 3:** Baseline-adjusted univariate linear regression analyses of predictors of echocardiographic changes following dual-chamber pacemaker implantation

Variable	LVEF (%)		RVGLS (%)		LAScd (%)	
	β (95% CI)	*P*-value	β (95% CI)	*P*-value	β (95% CI)	*P*-value
Pre-implantation value	–0.082 (–0.350 to 0.186)	0.541	0.550 (0.096 to 1.004)	0.019	–0.111 (–0.400 to 0.178)	0.438
Age (yr)	0.065 (–0.077 to 0.206)	0.364	–0.111 (–0.267 to 0.046)	0.160	–0.323 (–0.494 to –0.152)	< 0.001
Diabetes mellitus	–4.337 (–11.430 to 2.757)	0.224	–3.025 (–9.977 to 3.928)	0.380	–1.483 (–7.697 to 4.731)	0.629
Dyslipidemia	–0.790 (–7.739 to 6.159)	0.820	2.358 (–3.709 to 8.425)	0.433	2.460 (–3.011 to 7.932)	0.365
Hypertension	–3.154 (–9.605 to 3.297)	0.329	0.999 (–4.454 to 6.451)	0.710	1.760 (–3.230 to 6.751)	0.476
Chronic kidney disease	–1.173 (–9.754 to 7.409)	0.784	3.393 (–3.993 to 10.779)	0.355	–0.588 (–6.837 to 5.660)	0.848
High pacing burden (> 20%)	0.978 (–4.208 to 6.164)	0.705	–0.078 (–4.770 to 4.614)	0.937	2.166 (–2.122 to 6.454)	0.310

*CI* confidence interval, *LAScd* left atrial conduit strain, *LVEF* left ventricular ejection fraction, *RVGLS* right ventricular global longitudinal strain

### Changes in TR severity

**Fig. 2:**
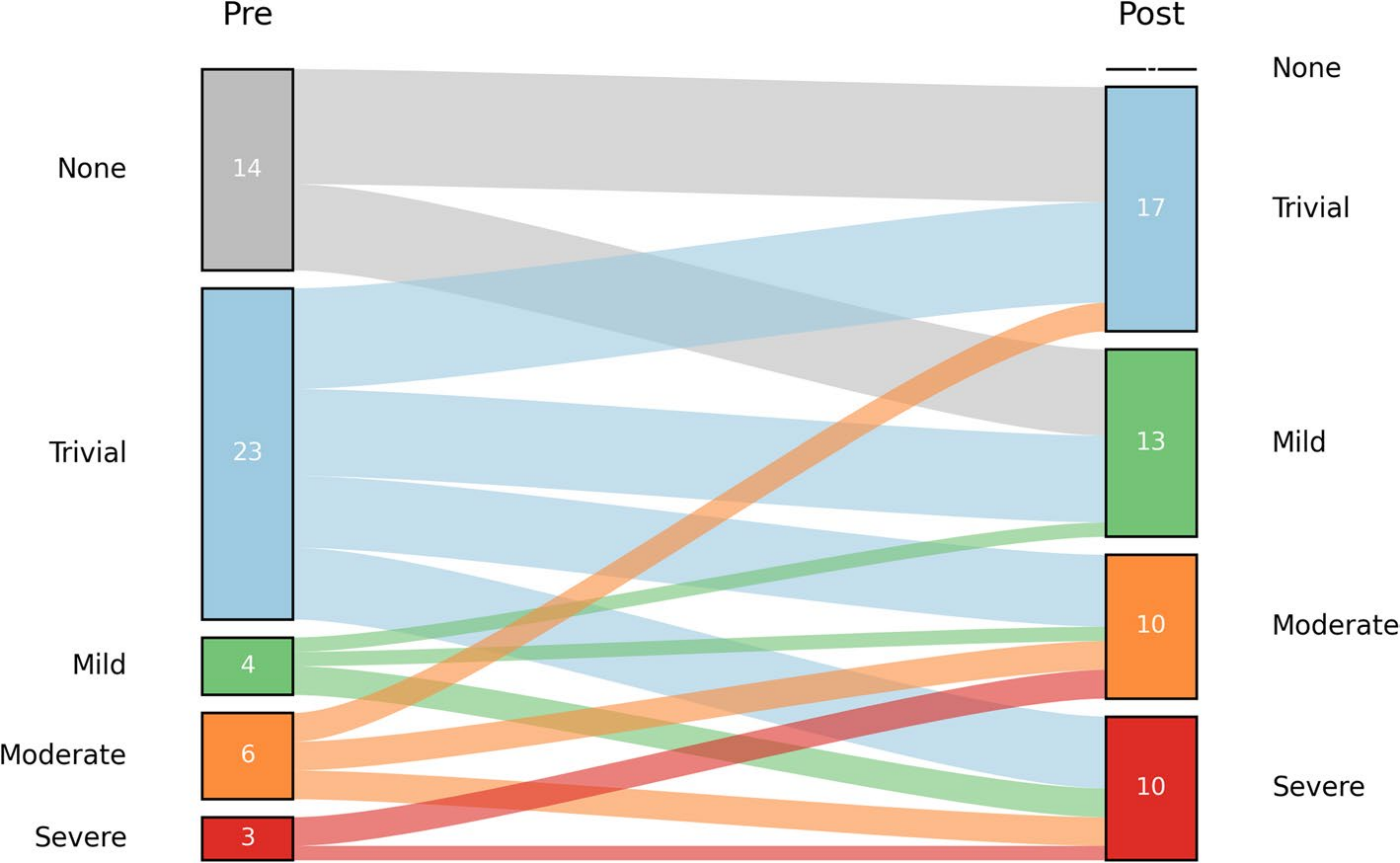
Sankey diagram illustrating patient-level transitions across five tricuspid regurgitation severity categories (none, trivial, mild, moderate, severe) from before to after implantation. The width of each flow represents the number of patients transitioning between categories

TR severity is summarized in a Sankey diagram (Fig. 2) and Table 2. Among patients with paired assessments (*n* = 50), transitions in TR severity demonstrated both progression and regression across categories (Table S2). Of the 14 patients with no TR at baseline, 8 (57.1%) progressed to trivial TR, and 6 (42.9%) progressed to mild TR at follow-up. Among the 23 patients with trivial TR at baseline, 7 (30.4%) remained trivial, 6 (26.1%) progressed to mild TR, 5 (21.7%) progressed to moderate TR, and 5 (21.7%) progressed to severe TR. Of the four patients with mild TR at baseline, one (25.0%) remained mild, one (25.0%) progressed to moderate TR, and two (50.0%) progressed to severe TR. Among the six patients with moderate TR at baseline, two (33.3%) improved to trivial TR, two (33.3%) remained moderate, and two (33.3%) progressed to severe TR. Of the three patients with severe TR at baseline, one (33.3%) remained severe, and two (66.7%) improved to moderate TR. However, when TR was analyzed as a paired ordinal variable across the five severity categories (none, trivial, mild, moderate, and severe), the overall shift in TR severity was not significant in Stuart-Maxwell testing following pacemaker implantation (χ^2^ = 24.94, *P* = 0.678).

### Changes in RV, LA, and strain-based parameters

**Fig. 3:**
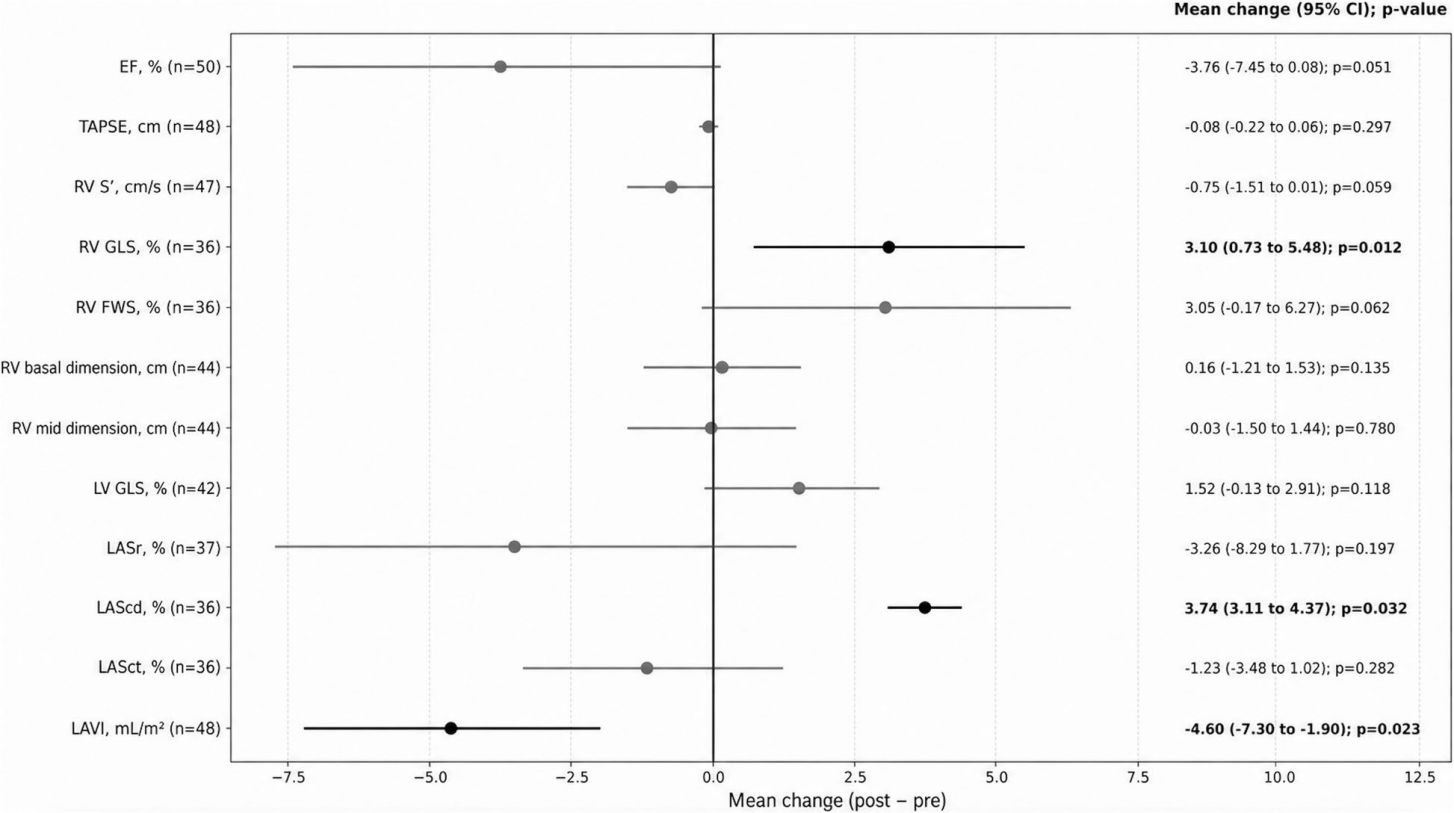
Forest plot demonstrating mean changes in echocardiographic parameters following dual-chamber pacemaker implantation. Points represent mean differences, and horizontal lines indicate 95% confidence intervals (CIs). EF, ejection fraction; LAScd, left atrial conduit strain; LASct, left atrial contractile strain; LASr, left atrial reservoir strain; LAVI, left atrial volume index; LVGLS, left ventricular global longitudinal strain; RV, right ventricular; RVFWS, right ventricular free wall strain; RVGLS, right ventricular global longitudinal strain; TAPSE, tricuspid annular plane systolic excursion

Overall, the forest plot illustrates a pattern of modest adverse changes in selected functional and deformation parameters, with only RVGLS and LAScd attaining statistical significance (Fig. 3). A summary of these outcomes can be found in Table 2. RV systolic function, assessed by TAPSE, did not change significantly following pacemaker implantation (mean change, − 0.08 cm; 95% CI, − 0.22 to 0.06; *P* = 0.297). RV S’ demonstrated a modest numerical reduction that did not reach statistical significance.

The strain-based measures demonstrated more pronounced changes. RVGLS worsened following pacemaker implantation (mean change, 3.10%; 95% CI, 0.73 to 5.48; *P* = 0.012), as did LAScd (mean change, 3.74%; 95% CI, 3.11 to 4.37; *P* = 0.032). Changes in LVGLS, RVFWS, LASr, and LASct were not statistically significant.

LAVI decreased significantly following pacemaker implantation (38.98 ± 17.16 mL/m^2^ vs. 34.38 ± 12.65 mL/m^2^, *P* = 0.023). However, LAVI and LAScd did not correlate significantly (Spearman *ρ* =  − 0.183, *P* = 0.265). Similarly, changes in LAVI before and after implantation did not correlate significantly with changes in LAScd over time (Spearman *ρ* = 0.160, *P* = 0.350).

In the baseline-adjusted univariate linear regression analyses, no baseline clinical variables were significantly associated with the change in LVEF following pacemaker implantation. Higher baseline RVGLS was associated with a greater decline in RVGLS over time (β = 0.550; 95% CI, 0.096 to 1.004; *P* = 0.019). No other baseline clinical variables (age, comorbidities, or pacing burden) were significantly associated with changes in RVGLS. On the other hand, increasing age was independently associated with a greater decline in LAScd (β =  − 0.323 per year; 95% CI, − 0.494 to − 0.152; *P* < 0.001). No other baseline clinical variables, including pacing burden, were associated with changes in LAScd. Notably, a high RV pacing burden (> 20%) was not significantly associated with changes in LVEF, RVGLS, or LAScd (Table 3).

## Discussion

To our knowledge, this is the first single-center study in Malaysia to specifically investigate echocardiographic outcomes after dual-chamber pacemaker implantation. Significantly, strain worsened, as evidenced by the reduction in RVGLS and LAScd. Although the EF trended toward a decrease, the change was not statistically significant. Furthermore, TR severity did not change after implantation.

Our finding of worsening strain, particularly RVGLS, is consistent with a recent study by Kamath et al. [13], which reported a decline in RVGLS, right atrial reservoir, and conduit strain 3 months after implantation. Our data extend those observations to a longer follow-up of 6 months, suggesting that dual-chamber pacing can be associated with early adverse changes in myocardial deformation. However, the observed reduction in RVGLS should be interpreted with caution. RVGLS incorporates both septal and free wall segments and might therefore be influenced by pacing-induced interventricular dyssynchrony. In patients with RV pacing, abnormal septal motion might not reflect intrinsic myocardial contractility, but rather altered electrical activation patterns. This could result in an apparent or pseudo-reduction in global strain despite relatively preserved RV systolic function [10, 14]. In contrast, RVFWS, which excludes the septal segments, did not demonstrate a statistically significant change in our patients. This discrepancy supports the current ASE/European Association of Cardiovascular Imaging recommendations that prioritize RVFWS over RVGLS when assessing RV function in patients with conduction abnormalities or pacing, in whom septal motion can be confounded by dyssynchrony [10]. Taken together, our findings highlight the importance of interpreting strain parameters within the context of underlying electrical activation and suggest that, following pacemaker implantation, RVFWS might provide a more physiologically representative measure of RV systolic function than RVGLS.

Although LVEF trended toward reduction (3.8%) after implantation, as reported in other studies [13, 15], the change was not statistically significant, perhaps because our small sample size limited our statistical power. In this study, we incorporated pacing burden into our analyses to better evaluate the mechanistic contribution of pacing to the observed LVEF. Approximately 42.1% of patients demonstrated a high RV pacing burden (> 20%). However, that burden was not significantly associated with changes in LVEF, RVGLS, or LAScd in the regression analyses, consistent with the findings of Riahi et al. [16]. Our findings suggest that the observed alterations in myocardial deformation cannot be solely attributed to the pacing burden. Whereas prior studies demonstrated that a high RV pacing burden can contribute to pacing-induced cardiomyopathy through electrical and mechanical dyssynchrony [17], our results highlight that the relationship between pacing and myocardial function is likely more complex than that, particularly in patients predominantly being treated for sick sinus syndrome, in whom intrinsic conduction can be intermittently preserved.

In this study, LAScd declined following pacemaker implantation. However, this finding should be interpreted in the context of LV-LA coupling, rather than intrinsic atrial pathology. LAScd is predominantly determined by early diastolic LV properties, including LV relaxation and suction, rather than atrial compliance or fibrosis alone [18, 19]. The observed association with aging is therefore likely to reflect progressive impairment in LV diastolic function, rather than primary atrial remodeling [20]. To further contextualize these findings, LAVI was assessed as a marker of structural remodeling. In our cohort, LAVI decreased significantly following pacemaker implantation. However, no significant correlation was observed between LAVI and LAScd at baseline or between their respective changes over time. This lack of association suggests a dissociation between the structural and functional atrial parameters. The reduction in LAVI might reflect changes in loading conditions, rather than true reverse remodeling of the LA [20]. Taken together, our findings support the interpretation that the observed reduction in LAScd reflects early functional impairment, likely driven by alterations in LV diastolic function, rather than established structural remodeling. This interpretation is also consistent with prior observations that strain-based indices are more sensitive than volumetric measures in detecting early diastolic dysfunction and that functional abnormalities can precede overt atrial enlargement [18, 21].

Several studies have reported TR progression following device implantation [13, 22, 23]. In our study, a directional trend toward worsening TR severity was observed at 6 months after procedure; however, it did not reach statistical significance. Furthermore, the RV basal and mid dimensions remained unchanged, suggesting the absence of significant volume overload in this cohort. Although transvalvular lead placement can interfere with tricuspid valve mechanics, the lack of significant TR progression and absence of RV dilation in our data do not support a volume overload mechanism for the observed changes in myocardial deformation. Instead, those changes might reflect alterations in ventricular activation and mechanics.

The chief limitation of this study is the small sample size, along with the smaller number of patients with both pre- and post-implantation echocardiographic data. This limited our ability to perform modeling analyses to identify variables that might predict some of our findings. Second, although our study offers longer-term follow-up results than previous reports, future studies should consider reporting follow-up data from up to 12 months to adequately assess longitudinal and temporal changes in cardiac function. Third, we used 2D echocardiography because it was part of the standard clinical practice at the time of this study. A useful follow-up study could implement 3D echocardiography to thoroughly assess both left and right heart function.

Fourth, TR severity was assessed qualitatively as part of routine clinical echocardiographic reporting, without systematic incorporation of quantitative or semiquantitative parameters such as vena contracta width, effective regurgitant orifice area, or hepatic vein flow patterns [11]. As a result, TR grading could be subject to intraobserver variability and limited precision, particularly in distinguishing between adjacent severity categories. Furthermore, the retrospective design of this study precluded the standardization of TR assessment across all patients, and variations in image quality and reporting practices might have influenced that classification. Although we reclassified TR severity into the ASE guideline–based categories for our analysis [11], the absence of uniform quantitative criteria could affect the accuracy of the severity transitions observed. Nonetheless, the paired study design, with each patient serving as their own control, mitigates this limitation by allowing us to evaluate directional changes in TR severity over time.

Fifth, although missing strain data were primarily attributable to suboptimal image quality, the image quality itself can be influenced by patient-related factors. Although we demonstrated no differences in baseline characteristics between patients with and without strain data, the possibility of selection bias cannot be excluded, which could limit the generalizability of the strain-based findings. Sixth, intraobserver reproducibility for the echocardiographic measurements was not formally assessed, which could limit the precision and reproducibility of the strain-derived parameters. Seventh, the underlying cardiac rhythm at the time of echocardiographic acquisition could not be reliably determined due to limited and variable-quality electrocardiogram traces during image acquisition. Because myocardial deformation parameters can differ between intrinsic and paced rhythms, this limitation represents a potential source of variability. The device-derived pacing burden and pacing mode were used as surrogate markers, but they might not fully capture beat-to-beat variations in ventricular activation.

## Conclusions

In this single-center, paired echocardiographic study, dual-chamber pacemaker implantation was associated with modest changes in ventricular and atrial function, including the worsening of selected strain-derived parameters. Although most echocardiographic measures remained stable, a subset of patients demonstrated a trend toward worsening severe TR following implantation. These findings support the role of strain imaging in post-implant surveillance and justify further studies to identify patients at risk of pacing-related cardiac dysfunction.

## Supplementary Information

Additional file 1: Table S1. Baseline characteristics were comparable between patients with and without available LVEF data, and between those with and without available strain data. Table S2. Transition matrix of tricuspid regurgitation severity from pre- to post-implantation (paired analysis, *n* = 50).

## Data Availability

Available with restricted access. The data that support the findings of this study are available from the corresponding author upon reasonable request.

## Abbreviations

ASE: American Society of Echocardiography
CI: Confidence interval
EF: Ejection fraction
GLS: Global longitudinal strain
LA: Left atrial
LAScd: Left atrial conduit strain
LASct: Left atrial contractile strain
LASr: Left atrial reservoir strain
LAVI: Left atrial volume index
LV: Left ventricular
LVEF: Left ventricular ejection fraction
LVGLS: Left ventricular global longitudinal strain
RV: Right ventricular
RVFWS: Right ventricular free wall strain
RVGLS: Right ventricular global longitudinal strain
TAPSE: Tricuspid annular plane systolic excursion
TR: Tricuspid regurgitation
