# Supplementary material for: Echocardiographic changes following dual-chamber pacemaker implantation at a Malaysian tertiary heart center
Source: J Cardiovasc Imaging. 2026 Jul 6;34:16. doi: 10.1186/s44348-026-00077-6 (PMC13335278; doi:10.1186/s44348-026-00077-6)
Supplement: Supplementary file 1 — Additional file 1: Table S1. Baseline characteristics were comparable between patients with and without available LVEF data, and between those with and without available strain data. Table S2. Transition matrix of tricuspid regurgitation severity from pre- to post-implantation (paired analysis, n = 50). [file 44348_2026_77_MOESM1_ESM.docx]

| Supplementary Table 1: Baseline characteristics were comparable between patients with and without available LVEF data, and between those with and without available strain data | | | | | | | | | | | | |
| --- | --- | --- | --- | --- | --- | --- | --- | --- | --- | --- | --- | --- |
| Outcome | EF | | P-value | TAPSE | | P-value | RV GLS | | P-value | LAScd | | P-value |
| Data missing | Yes  (50) | No  (7) |  | Yes  (48) | No  (9) |  | Yes  (36) | No  (21) |  | Yes  (36) | No  (21) |  |
| Variable |  |  |  |  |  |  |  |  |  |  |  |  |
| Age (year) | 59.5±21.0 | 44.8±16.1 | 0.20 | 61.0±19.0 | 55.8±18.1 | 0.36 | 59.9±11.0 | 61.3±26.1 | 0.89 | 59.5±18.0 | 46.9±29.1 | 0.44 |
| Gender (male) | 84.4 | 71.4 | 0.89 | 81.3 | 66.7 | 0.91 | 62.5 | 57.1 | 0.91 | 62.5 | 57.1 | 0.91 |
| Diabetes mellitus | 26.0 | 28.6 | 0.98 | 27.1 | 22.2 | 0.98 | 27.8 | 23.8 | 0.11 | 25.0 | 28.6 | 0.88 |
| Hypertension | 54.0 | 42.9 | 0.10 | 52.1 | 55.6 | 0.78 | 55.6 | 47.6 | 0.56 | 52.8 | 52.4 | 0.45 |
| Hypercholesterolemia | 24.0 | 28.6 | 0.56 | 25.0 | 22.2 | 0.86 | 26.0 | 23.8 | 0.56 | 19.4 | 33.3 | 0.09 |
| Chronic kidney disease | 8.0 | 14.3 | 0.06 | 8.3 | 11.1 | 0.10 | 8.3 | 9.5 | 0.38 | 8.3 | 9.5 | 0.89 |

Continuous variable reported as mean±S.D; categorical variables reported as %

| Supplementary Table 2: Transition matrix of tricuspid regurgitation severity from pre- to post-implantation (paired analysis, N = 50) | | | | | |
| --- | --- | --- | --- | --- | --- |
| Pre / Post | None | Trivial | Mild | Moderate | Severe |
| None | 0 | 8 | 6 | 0 | 0 |
| Trivial | 0 | 7 | 6 | 5 | 5 |
| Mild | 0 | 0 | 1 | 1 | 2 |
| Moderate | 0 | 2 | 0 | 2 | 2 |
| Severe | 0 | 0 | 0 | 2 | 1 |
